# Locoregional Combined With Systemic Therapies for Advanced Hepatocellular Carcinoma: An Inevitable Trend of Rapid Development

**DOI:** 10.3389/fmolb.2021.635243

**Published:** 2021-04-13

**Authors:** Xin Li, Yaxi Wang, Xin Ye, Ping Liang

**Affiliations:** ^1^ Department of Interventional Ultrasound, Chinese PLA General Hospital, Beijing, China; ^2^ Department of Ultrasound, The Affiliated Hospital of Inner Mongolia Medical University, Hohhot, China; ^3^ Department of Oncology, The First Affiliated Hospital of Shandong First Medical University and Shandong Provincial Qianfoshan Hospital, Shandong Lung Cancer Institute, Jinan, China

**Keywords:** hepatocellular carcinoma, tyrosine kinase inhibitors, immune checkpoint inhibitors, thermal ablation, transarterial chemoembolization

## Abstract

Despite the application of antiviral drugs and improved surveillance tools, the number of patients diagnosed with hepatocellular carcinoma (HCC) at an advanced stage and with a dismal prognosis is still on the rise. Systemic treatment with multiple multitargeted tyrosine kinase inhibitors (TKIs), such as sorafenib, has been a widely utilized approach for a decade. In addition, the use of a combination of TKIs with other types of compounds, including immune checkpoint inhibitors (ICIs) and antiangiogenic inhibitors, has shown efficacy in treating advanced HCC. However, the presence of intolerable adverse events, low disease response and control rates, and relative short overall survival of such combinatory therapies makes novel or optimized therapies for advance HCC urgently needed. Locoregional therapy (transarterial chemoembolization, and thermal ablation) can destroy primary tumors and decrease tumor burden and is widely used for HCC management. This type of treatment modality can result in local hypoxia and increased vascular permeability, inducing immunogenic effects by releasing tumor antigens from dying cancer cells and producing damage-associated molecular patterns that facilitate antiangiogenic therapy and antitumor immunity. The combination of systemic and locoregional therapies may further produce synergistic effects without overlapping toxicity that can improve prognoses for advanced HCC. In preliminary studies, several combinations of therapeutic modes exhibited promising levels of safety, feasibility, and antitumor effects in a clinical setting and have, thus, garnered much attention. This review aims to provide a comprehensive, up-to-date overview of the underlying mechanisms of combined systemic and locoregional therapies in the treatment of advanced HCC, commenting on both their current status and future direction.

## Introduction

Liver cancer is the sixth most commonly diagnosed cancer and the fourth leading cause of cancer-related death worldwide, representing a highly fatal tumor with dismal prognosis (GBD 2015 Mortality and Causes of Death Collaborators, 2016; Bray et al., 2018; Siegel et al., 2020). The 5-year overall survival (OS) of liver cancer is 18%, making it the second most lethal tumor after pancreatic cancer, which has an OS of 9% (Villanueva, 2019). Additionally, most cases are diagnosed at the advanced stage and with an incidence-to-mortality ratio that approaches one (Singal et al., 2020). About 75–85% of primary liver cancers are hepatocellular carcinoma (HCC), which has become a major health problem worldwide (Bray et al., 2018).

Because of improved tools for screening for cirrhosis and HCC, early-stage HCC can potentially be cured through surgical resection, ablation, or liver transplantation. But, the high recurrence and metastasis rates after such radical treatments still affect the long-term survival (El Dika et al., 2021). HCC patients experiencing recurrence and metastasis have entered into advanced HCC stages, and there is no current standard and effective treatment. The majority of HCC cases typically develop in a background of chronic liver disease resulting from hepatitis B or C virus (HBV or HCV) infection, alcohol abuse, aflatoxin B exposure, and metabolic diseases such as obesity, hemochromatosis, and diabetes (Craig et al., 2020). The application of HBV vaccines and antiviral drugs is likely to change this etiological landscape, but the incidence of HCC is still increasing due to alcohol abuse and nonalcoholic fatty liver disease. Changes in the etiological landscape of HCC contribute to the high molecular heterogeneity of HCC and affect the selection of treatment options, even the prognosis following treatment (Kulik and El-Serag, 2019). Chronic inflammation and subsequent cirrhosis can induce the development of HCC, which resulting in increased tumor immunogenicity. Because chronic inflammation in hepatitis could be recognized by the immune system, the immune cells’ aggregation could increase the number of immune cells. Once HCC cells are recognized by the immune system, they are more vulnerable to be attacked. The liver itself is an immunological organ; however, additional evidence has revealed immune system suppression in liver tumor microenvironments, leading to immune system resistance of HCC (Ghavimi et al., 2020; Lee et al., 2020). All these influences on the etiology and pathogenesis of HCC make the treatment of HCC difficult and uncertain.

Around 70–80% of HCC patients are initially diagnosed at an advanced HCC stage according to the widely applied Barcelona Clinic of Liver Cancer (BCLC) staging system. The BCLC staging system subdivides patients based on tumor burden, the degree of liver dysfunction, and liver performance status, which affect the efficacy of treatments (Forner et al., 2018; Bouattour et al., 2019). Systemic treatment with several multitargeted tyrosine kinase inhibitors (TKIs), including sorafenib, has been widely utilized for decades. Chemical agents like TKIs, as well as immune checkpoint inhibitors (ICIs) and antiangiogenic inhibitors, have proven effective in treating advanced HCC (Raybould and Sanoff, 2020). However, due to intolerable adverse events (AEs), low disease response rates, low disease control rates (DCRs), and relative short OS, novel and optimized therapies for advanced HCC are urgently needed. How to effectively help these advanced HCC patients is a challenging clinical problem and requires urgent management.

Locoregional therapy (thermal ablation (TA) and transarterial chemoembolization (TACE)) can destroy primary tumors and decrease the tumor burden and is widely used for HCC management. TA has been established as a first-line therapy for early-stage HCC, and TACE is the only recommended option available for advanced HCC (Forner et al., 2018; Villanueva, 2019). However, TA and TACE are also widely used in other contexts, contributing to promising outcomes for select patients with advanced-stage HCC, especially in China. In addition, more than 90% of patients receiving TACE have shown benefits and an improved prognosis (Park et al., 2015; Xie et al., 2020). In addition, patients could receive routine systemic treatments after TA or TACE as long as liver function and performance remain sufficient (Roderburg et al., 2020). Combinations of locoregional and systemic therapies have, thus, already been administrated in clinical settings. In China, the combination of TACE with other locoregional therapies such as ablation or with systemic therapies such as sorafenib is encouraged in select patients based on the latest Chinese clinical guidelines (Xie et al., 2020).

Based on the abovementioned information, combining systemic with locoregional therapies based on a multidisciplinary treatment approach is key to creating successful outcomes in patients with advanced HCC. This review, thus, aims to provide a comprehensive, up-to-date overview of the underlying mechanisms of combined systemic and locoregional therapies in the treatment of advanced HCC and updates on the current status and future direction of this approach.

## Locoregional Therapies

Although locoregional therapies are recommended for patients with HCC in BCLC stage 0, A, and B, selection of one of the available approaches should take into account several parameters that go beyond these general recommendations in real-world clinical practices, including tumor characteristics, patient-specific factors, treatment availability, and local expertise availability. The pros and cons of different locoregional therapies should be comprehensively evaluated in order to help select the right treatment for each patient.

### Thermal Ablation

TA destroys tumors through direct and indirect mechanisms (Nikfarjam et al., 2005). Ablated lesions can be thought of as having three zones: the central zone, the peripheral or transitional zone of sublethal hyperthermia, and the zone formed by the surrounding tissue that is unaffected by ablation (Ahmed et al., 2011). The central zone undergoes ablation-induced coagulative necrosis, a direct mechanism. Effects in the peripheral or transitional zone can be considered indirect mechanisms occurring mainly from thermal conduction, which may have immunogenic effects due to the release of tumor antigens and damage-associated molecular patterns from dying cancer cells. Both preclinical and clinical studies have led to the proposal of mechanisms underlying transitional zone effects that include ischemia, ischemia-reperfusion injuries, the release of lysosomal contents and cytokine, and the further stimulation of an immune response (Fajardo et al., 1980; Nikfarjam et al., 2005). Indirect mechanisms caused by TA have also formed a basis for antiangiogenic and antitumor immunity therapies (Chu and Dupuy, 2014).

TA is a well-established form of local cancer treatment and includes radiofrequency ablation (RFA), microwave ablation (MWA), cryoablation (CA), and irreversible electroporation (IER). Percutaneous ablation therapies focus on image-guided (US, CT, MRI, or C-arm) destruction of tumor tissues through the direct application of either chemical- or energy-based treatments, with the benefit of allowing for curative, palliative, or downstaging intent. Currently, the most commonly used forms of TA, and the main focus of this article, are RFA and MWA. One limitation of RFA is that it is highly risky when tumors are in close proximity to the liver capsule or other critical structures such as vasculature, which can make them susceptible to the heat-sink effect. Many studies have also shown that HCC lesions treated by RFA with perivascular cells have not been effectively ablated, which would increase the risk of local recurrence (Bhardwaj et al., 2009). In many liver-cancer-treating institutions, RFA has been replaced by MWA, which involves faster heating and allows for the treatment of lesions closer to vessels due to its lower heat-sink effect (Liang et al., 2013; Yu et al., 2017). In addition, one study showed that MWA exhibits potential superiority for the treatment of larger HCCs (Poulou et al., 2015), although the survival probability for MWA has been shown to be the greatest for lesions less than 4 cm (Liang et al., 2005). However, data are lacking on whether RFA or MWA provide better OS; thus, both techniques can be considered equally effective (Andreano and Brace, 2013; Yu et al., 2017).

TAs are optimal for patients with small HCCs made up of up to three lesions with a maximum of 3 cm size, each falling into Child–Pugh (CP) class A or B (Shiina et al., 2018). In one study, an OS of 74.2% was achieved in the patients followed up for 10 years after ablation (Cho et al., 2009). In this case, prognostic factors considered for OS included local tumor progression (LTP), CP class, platelet levels, intrahepatic distant recurrence, aggressive intrasegmental recurrence, and extrahepatic metastatic diseases (Cho et al., 2009). Currently, the recommended ablative margin (AM) that can decrease the LTP is 0.5–1.0 cm away from tumors (Ahmed et al., 2014). In addition, the use of multiple antennas during a single treatment can increase the treatment field and allow for the more effective treatment of larger lesions (Wright et al., 2003). Finally, combinations of therapies, such as TACE followed by MWA, have shown favorable outcomes for larger lesions and are often used for lesions not susceptible to treatment with just a single modality (Xu et al., 2013; Zhang et al., 2018).

Due to the application of three-dimensional (3D) visual surgical planning systems, ablative therapies have been increasingly used for larger and high-risk-location tumors (Li et al., 2017; Fang et al., 2020). In a comparable study, 223 liver tumors with mean diameters of 5.0 ± 1.5 cm were divided into 3D and 2D planning groups. Success rates of the first ablation were higher for the 3D planning group compared with those in 2D planning group (95.0% vs. 85.7%, *p* <0.05), and the LTP rate of the 3D planning group was lower than that of the 2D planning group (16.5% vs. 41.2%, *p* <0.05). 3D visual surgical planning systems, thus, appear to improve ablation precision, resulting in less LTP and higher AMs for patients with HCC lesions larger than 3 cm in diameter. However, no statistical differences were observed between the two groups in OS and RFS, which were affected by the comprehensive fact that larger tumors are more prone to developing satellites and vessels that feed the tumor (An et al., 2020). In another study, RFA combined with TACE assisted by a 3D visualization ablation planning system provided optimal clinical efficiency for HCC in challenging locations and was verified as a highly safe treatment modality (Huang et al., 2020). Although no statistically significant differences were detected in OS between the study group and the control group, however, the 1-, 2-, and 3-year LTP rates of the study group showed superior local control rates compared to the control group.

### Transarterial Chemoembolization

Since HCCs are solely supplied by the hepatic artery, transarterial treatments have proven to be extremely effective in delivering targeted embolic therapies to tumors while preserving and minimizing exposure to the surrounding liver parenchyma, especially when performed in an extremely selective manner (Breedis and Young, 1954; Kerbel, 2008; Pillai et al., 2020). TACE is the most widely used therapeutic intervention for patients with intermediate-stage HCC (Palmer et al., 2020). There are four types of embolization used for HCC treatment in clinic: bland embolization, conventional TACE, drug-eluting bead chemoembolization (DEB-TACE), and TARE (Kritzinger et al., 2013; Kloeckner et al., 2020). A recent meta-analysis of 55 randomized controlled studies did not show any significant survival benefit provided by cTACE, DEB-TACE, or TARE compared to TAE. There were 55 randomized control trials (12 direct comparisons) involving 5,763 patients with preserved liver function and unresectable advanced-stage HCC. Also, the results indicated that the OS was 18.1 months after treatment with TACE, 20.6 months with DEB-TACE, 20.8 months with bland TAE, 30.1 months with TACE plus external radiotherapy, 33.3 months with TACE plus liver ablation, and 13.9 months with the control treatment. All embolization strategies caused significant survival gains over control treatments. However, TACE, DEB-TACE, and TARE with adjuvant systemic agents did not provide any survival benefit over bland TAE alone (Katsanos et al., 2017). According to EASL and AASLD guidelines, TACE is a recommended first-line therapy for intermediate BCLC B HCC and has been recommended for the treatment of CNLC stage Ib to IIIb HCC by the 2019 version of the Chinese clinical guidelines for HCC management (European Association for the Study of the Liver, 2018; Marrero et al., 2018; Zhou et al., 2018; Department of Medical Administration, N.H., 2020).

Studies reported that the median OS after TACE in a group of patients with intermediate-stage HCC varied from 2.5 to 4 years depending on how strictly the inclusion criteria were followed (Burrel et al., 2012; Golfieri et al., 2014). BCLC stage B describes a heterogeneous group of patients with different degrees of liver function impairment (Llovet et al., 1999; European Association for the Study of the Liver, 2018). Subclassifications for BCLC stage B have been proposed; however, none have been widely adopted in the clinic (Bolondi et al., 2012; Kudo et al., 2015). Another study reported that TACE was applied to BCLC stage C HCC patients and extended survival time from 18.5 to 20.4 months. This was comparable to results of the application of systemic therapies involving first- and second-line treatments, which prolonged survival from 8 to 13 months (Li et al., 2018). In addition, the presence of portal vein tumor thrombus, maximum tumor size, CP score, and AFP levels helped predict survival time of BCLC stage C HCC patients. In summary, careful patient selection remains challenging in clinical practices. It is, thus, essential to discuss the indications for each potential treatment with a multidisciplinary board consisting of experienced investigators from radiology, interventional, oncology, hepatobiliary surgery, and digestive departments.

TACE is widely used for HCC stages ranging from BCLC 0 to C. For BCLC stage 0, TACE can be an alternative treatment for patients unsuitable for ablation, hepatic resection, or liver transplantation. However, although no statistically significant difference in OS was observed between groups of stage 0 HCC patients receiving either TACE or RFA, RFA treatment led to better tumor responses and delayed tumor progression. TACE may, thus, be considered a viable alternative treatment for treating single HCCs that are 2 cm or smaller when RFA is not feasible (Kim et al., 2014). In patients with BCLC stage A, TACE plus RFA provides better local control of tumors control than RFA alone. In addition, TACE can serve as bridge therapy for patients awaiting liver transplantation (Yao et al., 2008; Bouchard-Fortier et al., 2011). Importantly, TACE is a standard palliative therapy for BCLC B stage and can provide additional survival benefits compared to other supportive care options (Han and Kim, 2015).

Advanced HCC often exhibits larger or infiltrative tumors bridging two or more liver Couinaud Bismuth segments that cannot be managed by monotherapies due to low rates of complete responses (Kothary et al., 2015). In contrast, single TACE procedures often cause extensive tumor necrosis and provide tumor control. However, the degree of hypervascularity, an independent prognostic factor useful after TACE, and the infiltrative nature of tumors without well-defined borders were not included in any related guidelines (Katyal et al., 2000; Hu et al., 2011), and initial trials were designed to implement the first round of two TACE procedures within 1–2 months. A subsequent study showed that such an approach increases the response rate and that treatment repetition should be a common practice (Georgiades et al., 2012) and that subsequent ablations are more effective as reflected by the minimization of heat loss via convection. Moreover, TACE combined with ablation has synergistic cytotoxic effects toward HCC, with combination therapy providing higher efficacy than ablation alone (Kim et al., 2019; Patidar et al., 2019; Li et al., 2020).

Although a repetition strategy for TACE use can be applied, the nature of this palliative therapy cannot be changed. TACE can occlude the main arteries supplying a tumor while chemotherapy drugs destroy tumor cells. The hypoxic environments created by TACE further induced neoangiogenesis by stimulating vascular endothelial growth factor (VEGF) production and other angiogenic pathways, promoting revascularization and growth of residual viable tumors and potentially leading to tumor recurrence and metastasis (Wang et al., 2008; Fernández et al., 2009; Llovet et al., 2016). Many studies have been conducted that combined TACE with systemic antiangiogenic agents based on their established activities against advanced HCC, with previous confirmation of the safety of TACE combined with sorafenib. The GIDEON trial examined BCLC stage A–C HCC patients treated with either sorafenib alone or in combination with TACE either concomitantly or sequentially. No significant difference was detected in the incidence of AEs, while the OS was 12.7months in TACE patients, 9.2months in non-TACE patients, 21.6 months in concomitant-TACE patients, and 9.7months in non-concomitant-TACE patients (Geschwind et al., 2016). These results indicated that the combination of sorafenib and TACE had synergistic clinical effects with no overlapping effects in AEs, thus showing that combining TACE with systemic antiangiogenic agents is a safe, feasible, and efficient approach for treating HCC (Lencioni et al., 2014; Yao et al., 2015; Peng et al., 2019). In addition, the results of the latest TACTICS trial from Japan indicate that TACE plus sorafenib significantly improves PFS compared to TACE treatment alone in patients with unresectable HCC. AEs were consistent with those of previous TACE combination trials. A significant difference was detected in PFS, with 25.2m in TACE plus sorafenib compared with 13.5m in TACE alone. While the OS was not analyzed because only 73.6% of the OS events were recorded, the 1- and 2-year OS rates for the TACE plus sorafenib group were 96.2 and 82.7%, respectively, compared to 1- and 2-year OS rates of 77.2 and 64.6% in the TACE alone group, with no unexpected toxicities (Kudo et al., 2020).

## Systemic Therapies

Systemic therapies are currently recommended as the standard treatment for advanced HCC. Results of the SHARP trial conducted in 2008 suggested that the median OS and time to radiologic progression were almost 3 months longer for patients treated with sorafenib than for those given a placebo (10.7 vs. 7.9 m and 5.5vs. 2.8 m, respectively). In 2009, another randomized trial in the Asia–Pacific region achieved similar promising results with a better median OS in the sorafenib-treated group compared with the placebo-treated control group (6.5 vs. 4.2 m, respectively) (Cheng et al., 2009). In addition, sorafenib is considered a standard systemic treatment for HCC (Llovet et al., 2008). Such approval of sorafenib is a significant step forward in the management of advanced HCC and ushered forward several important concepts in drug development.

In the past decade, no targeted therapy has proven to have a higher clinically significant OS benefit for advanced HCC than sorafenib. However, in 2018, a phase III noninferiority trial showed better clinical effects from treatment with lenvatinib compared to sorafenib, including better OS (13.6 vs. 12.3 m), PFS (7.4 vs. 3.7 m), and time to progression (8.9 vs. 3.7 m) (Kudo et al., 2018). At present, lenvatinib and sorafenib are both recommended as first-line standard treatments. Both drugs are only applied in patients with good liver performance, ECOG of 0 or 1, and good hepatic function, with overall response rates (ORRs) of 10–20%. Similar AEs occurred with both treatments, including palmar–plantar skin reactions, fatigue, diarrhea, nausea, hypertension, bleeding, and weight loss (da Fonseca et al., 2020).

When HCC patients are refractory to or intolerant of first-line drugs, they are sometimes responsive to various second-line drugs, including regorafenib, cabozantinib, and ramucirumab, which have been recently approved by the FDA. Additionally, in China, apatinib has been recommended as a second-line treatment based on class IA evidence (Ni and Ye, 2019; Villanueva, 2019; Xie et al., 2020). Regorafenib was the first agent to improve survival as a second-line treatment compared to a placebo (10.6 vs. 7.8 m) (Bruix et al., 2017). In the phase III CELESTIAL trial, cabozantinib improved median OS compared to a placebo (10.2 vs. 8.0 m, respectively) and improved the median PFS compared to the placebo group (5.2 vs. 1.9 m, respectively) (Abou-Alfa et al., 2018). In the REACH-2 trial, ramucirumab increased OS to 8.5 m compared to 7.3 m for placebo treatment, accompanied by an improvement in PFS from 1.6 to 2.8 m (Zhu et al., 2019). At the 2020 ASCO annual meeting, the AHELP study was presented that studied the use of apatinib as a second-line treatment in Chinese patients with advanced HCC, yielding an OS of 9.8 m, with phase III trial results to come. Certain drawbacks are apparent upon treatment with these second-line target drugs, as the ORR ranges from 5–10% and high-grade AEs are more common, which may affect their applications in the clinic. For this aspect of treatments especially, novel approaches are urgently needed.

The liver itself is an immunological organ; however, its immunity when HCC is present is generally compromised. HCC often develops in association with cirrhosis. In the liver, blood typically enters from the portal vein and hepatic artery and mixes in the hepatic sinusoids, while in the presence of cirrhosis, blood flow decreases and enables antigens from the gut to thoroughly contact immune cells (Ringelhan et al., 2018). Liver sinusoidal endothelial cells are exposed to significant amounts of bacterial antigens from portal circulation and act as antigen-presenting cells to regulate the immunogenicity of the liver microenvironment (Jenne and Kubes, 2013). As a result, liver sinusoidal endothelial cells increase differentiation of Treg cells (Doherty, 2016), upregulate immunosuppressive cytokines (such as interleukin (IL)-10 and transforming growth factor-β (TGF-β)), increase expression of coinhibitory molecules (such as programmed cell death-1 (PD-1) and cytotoxic T-lymphocyte-associated antigen-4 (CTLA-4)) (Joyce and Fearon, 2015), and impede the immune surveillance of oncogenic development and progression. Such an immunosuppressive microenvironment, thus, not only favors the occurrence, growth, and progression of malignant cells but also provides for an alternative therapeutic regimen in the correction of this abnormal intrahepatic immunogenic status (Zhang et al., 2020).

Nivolumab is a monoclonal antibody targeting the negative immunoregulatory human cell surface receptor PD-1. A large phase I/II study of nivolumab (CheckMate 040) was conducted including 262 patients with HCC with or without previous exposure to sorafenib. These patients showed an ORR of 14% by RECIST (18% by mRECIST), with a median duration of response of 17 m. The median OS for second-line therapy was 15.6 m. Based on these encouraging results, the FDA granted accelerated approval to nivolumab for patients with advanced-stage HCC who had previously been treated with sorafenib in 2018 (El-Khoueiry et al., 2017), thus heralding an era of immunotherapy for HCC. Subsequently, pembrolizumab, another anti-PD-1 monoclonal antibody, has received accelerated approval by the FDA for patients with advanced-stage HCC as a second-line option. The KEYNOTE-224 trial showed an ORR of 17% (RECIST v1.1), PFS of 4.9 m, and median OS of 12.9 m, which was comparable to results with nivolumab (Zhu et al., 2018). Unfortunately, in the phrase III clinical trials of nivolumab and pembrolizumab for advanced HCC, the results failed to meet the primary endpoints for both OS and PFS when compared to placebo treatment and optimal supportive care (Finn et al., 2020). Therefore, strategies utilizing combination therapies that can increase the number of responders are being pursued.

Camrelizumab (SHR-1210) is an anti-PD-1 antibody originating from China and was the subject of a multicenter, open-label, parallel-group, randomized, phase II trial performed. An ORR of 14.7% was achieved, with an OS at 6 months of 74.4%. All these results suggest that camrelizumab exhibits antitumor activity in pretreated Chinese patients with advanced HCC, causes manageable toxicity, and is a potential new treatment option for these patients. The phase III trial of camrelizumab is currently underway in China (Qin et al., 2020).

Tremelimumab as a monotherapy has been investigated for HCC patients, with promising trial results including an ORR of 17.6% and a median time to progression of 6.48 m (Sangro et al., 2013). In the clinic, tremelimumab has often been used for nonresettable HCC patients in conjunction with interventional procedures such as radiofrequency ablation, TACE, and cryoablation. In an initial study of tremelimumab in combination with ablation, the median PFS was 7.4 m and the OS was 12.3 m. These results suggest that combination therapies are viable potential new treatments for patients with advanced HCC (Duffy et al., 2017).

For all immunotherapies examined, treatment-related AEs consisted of manageable toxicities. The ORR of the immunotherapy drugs was less than 30% as monotherapies; thus, their application in combination therapies is anticipated (Li et al., 2019).

## Combination Therapies

TACE combined with ablation and applied to intermediate-stage HCC commonly achieves radical effects. Although systemic therapy is the only option recommended for advanced HCC with larger and multiple tumors and hypervascularity, TACE can be performed as an effective adjunctive therapy for controlling tumor progression and improving prognoses. TACE in combination with ablative or systemic therapies was discussed previously in the TACE section. In this section, combination therapies of immunotherapies, immunotherapy with antiangiogenesis inhibitors, and locoregional treatments with immunotherapies and antiangiogenesis inhibitors are mainly discussed.

### Combinations of PD-1 and CTLA-4 Antibodies

On March 10, 2020, the FDA granted accelerated approval for the combined use of nivolumab, a PD-1 inhibitor, plus ipilimumab, a CTLA-4 inhibitor, for the treatment of HCC patients who were previously treated with sorafenib. The FDA’s approval is based on cohort findings from the phase I/II CheckMate-040 trial (NCT01658878), which demonstrated an ORR of 33% (95% CI, 20–48) in patients who received the nivolumab and ipilimumab combination. Additionally, the DOR ranged from 4.6 to 30.5 m, with 88% of responses lasting at least 6 months, 56% lasting at least 12 months, and 31% of responses lasting 24 months or longer. A blinded independent central review using the modified Response Evaluation Criteria in Solid Tumors (RECIST) yielded an ORR at 35%, with a CR reported in 12% of patients and a PR in 22%. The complete experimental results were published in *JAMA Oncol* on Oct 1, 2020 (Yau et al., 2020), and it was concluded that nivolumab plus ipilimumab had manageable safety, a promising objective response rate, and durable responses, with the arm A regimen (4 doses nivolumab (1 mg/kg) plus ipilimumab (3 mg/kg) every 3 weeks and then nivolumab (240 mg/kg) every 2 weeks) receiving accelerated approval in the US based on these results. An investigation of this combination is under way as a first-line therapy for patients with HCC (NCT04039607).

### Combination of Immune Checkpoint Inhibitors and Anti-VEGF Therapy

#### Atezolizumab Plus Bevacizumab

Anti-VEGF therapies can reduce VEGF-mediated immunosuppression within the tumor and its microenvironment and may enhance anti-PD-1/PD-L1 efficacy by reversing VEGF-mediated immunosuppression and promoting tumor T-cell infiltration (Hegde et al., 2018). Following the initial encouraging results from a phase 1b study of atezolizumab plus bevacizumab in patients with untreated advanced HCC demonstrated acceptable tolerance and promising antitumor activity, with an ORR of 36% and a median PFS of 7 months, as described at the ESMO Annual Meeting 2019. Next, a global, open-label, phase III randomized trial compared the safety and efficacy of atezolizumab plus bevacizumab vs. sorafenib in patients with advanced HCC who had not received prior systemic therapies (Finn et al., 2020). The results showed that the OS at 12 months was 67.2% with atezolizumab-bevacizumab and 54.6% with sorafenib, with median PFS lengths of 6.8 and 4.3 m, respectively. AEs occurred for each group with no significant differences. Atezolizumab combined with bevacizumab, thus, resulted in better OS and PFS outcomes than sorafenib in patients with unresectable HCC, potentially representing a solid foundation for approval of atezolizumab plus bevacizumab as a new standard first-line therapy for unresectable HCC by the FDA. Based on this study, Qin proposed that concurrent PD-L1 and VEGF inhibition may be effective in reducing HCC recurrence by creating a more favorable immune microenvironment, thereby enhancing anticancer immunity. To test this hypothesis, the IMbrave 050 study was designed to evaluate the use of atezolizumab plus bevacizumab for treating patients with HCC having a high risk of recurrence following curative resection or ablation. Enrollment began in December 2019, and the estimated study completion date is March 2023 (Hack et al., 2020).

#### Camrelizumab Plus Apatinib

Treatment with both camrelizumab and apatinib, a tyrosine kinase inhibitor selectively acting on VEGF-2, is currently being investigated (Okusaka and Ikeda, 2018). A phase 1 trial from China was completed in 2018 and showed acceptable tolerance of this combination, with ORR and DCR values of 50.0 and 93.8%, respectively. The 6 m PFS rate in patients receiving 250 mg of apatinib was 51.3%, the 9-month PFS rate was 41.0%, and the median OS was not reached (Xu et al., 2019). This regimen has been examined in a phase II trial and is currently undergoing evaluation in comparison with sorafenib in an ongoing phase III trial in a first-line setting for advanced HCC (NCT03764293) (Finn and Zhu, 2020).

#### Locoregional Therapy Plus Immune Checkpoint Inhibitors With Anti-VEGF Therapy

For advanced cases of HCC, the combination of systemic therapies with immunotherapies is gaining broader enthusiasm worldwide in clinical practices. Monoimmunotherapies have been confirmed to have no additional effects in phase III studies. However, combinations of immunotherapies and antiangiogenesis inhibitors have recently been shown to have superior clinical effects. Given the increasing numbers of active agents and combinations available for systemic therapies, the role of these combinatorial therapies needs to be explored in other tumor stages. Ablation and TACE can then be used at times when no systemic therapies are available. Patients with intermediate-stage HCC with high tumor burdens may be better candidates for combined systemic and locoregional therapies. However, randomized prospective trials are needed to clarify these efficacy questions.

In advanced HCC with BCLC stage C, TACE with sorafenib may prolong survival and delay disease progression. In one trial, the median OS and PFS were 22 and 8 m, respectively, for a group treated with TACE plus sorafenib, and 18 and 6 m, respectively, in a group treated only with sorafenib (Wu et al., 2017). The basis for this effect is that VEGF levels increase after TACE, suggesting that pharmacological intervention impairs VEGF signaling and inhibits tumor recurrence and metastasis. In turn, sorafenib suppresses VEGF signaling by inhibiting VEGFRs, which is expected to enhance the efficacy of TACE by inhibiting angiogenesis and promoting tumor apoptosis (Strebel and Dufour, 2008). Locoregional therapy and systemic therapy are, thus, good treatment partners. The effect of these good partners was shown in a study by Fan, who revealed that a combination of apatinib, TACE, and MWA is safe and effective for BCLC C HCC patients. Patients in the combination therapy group had significantly longer median PFS and OS values than those in the TACE alone group, with a PFS of 4.5 vs. 2.1 m, respecitvely, and an OS of 24.4 vs. 5.4 m, respectively. In addition, the AEs can be managed by adjusting the apatinib dosage (Shuanggang et al., 2020).

Immunotherapy may also be a perfect partner for locoregional therapy. The release of tumor-associated antigens during all types of locoregional therapies may stimulate immune responses, ideally leading to a synergistic effect by both therapies. Proof-of-concept experiments have been conducted with tremelimumab in combination with ablation. In 2017, when tremelimumab combined with ablation was applied to patients with advanced HCC, 26.3% (5/19) achieved a confirmed partial response, with a median time to tumor progression of 7.4 m and an OS of 12 m. This study suggests that the killing of tumors by direct methods (ablation) can activate the immune system, which potentially can recognize and kill the remaining cancer. In addition, drugs classified as ICIs could enhance this effect (Duffy et al., 2017).

**FIGURE 1:**
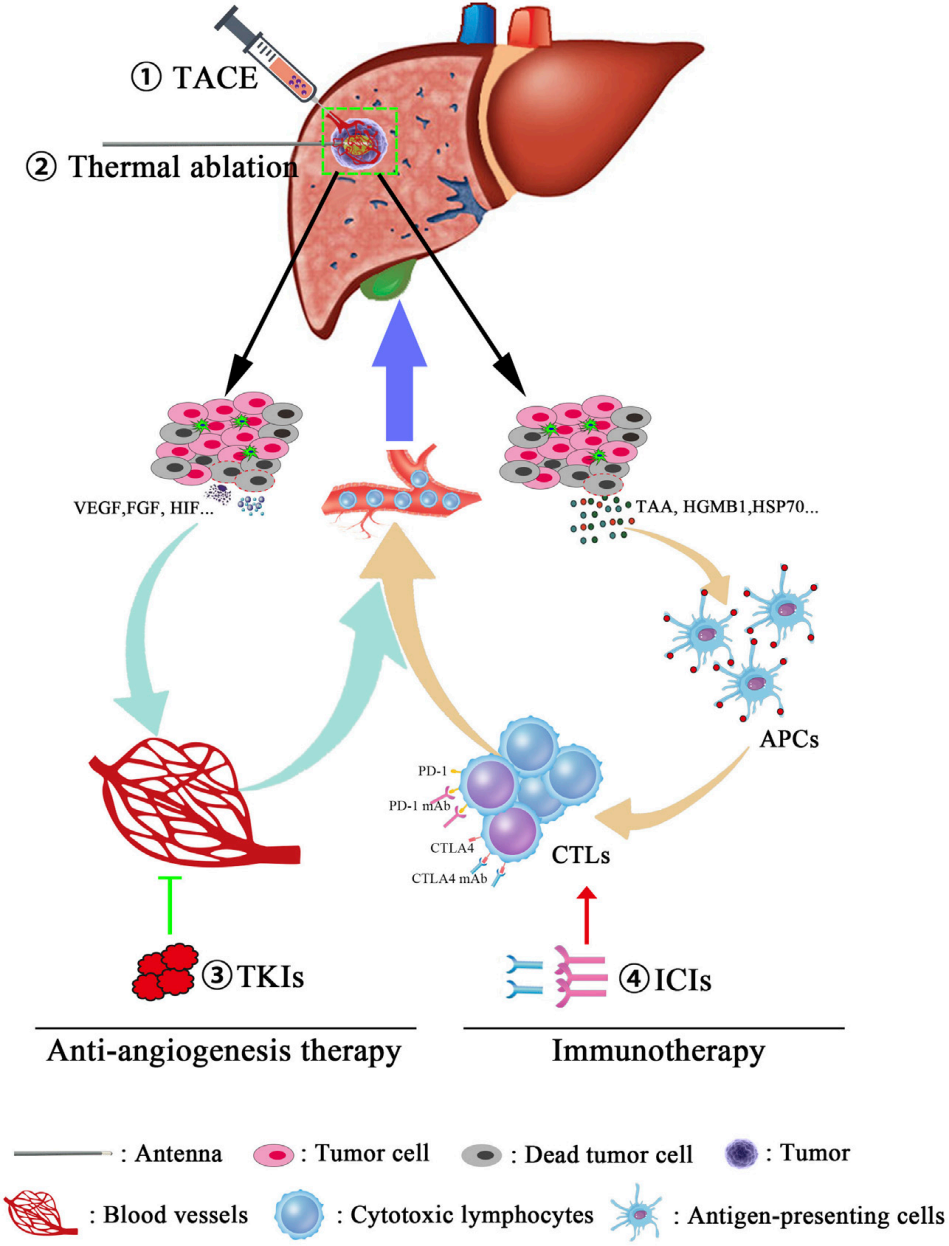
Combination therapies for the treatment of advanced hepatocellular carcinoma. Multitreatment modalities were used for managing tumor progress, and the detailed procedures are also described as follows: First, for the purpose of tumor burden reduction, the cancerous blood supply was obstructed using transcatheter arterial chemoembolization (TACE). Then, microwave ablation will further promote the tumor destruction in a manner of inducing an irreversible heat injury. Third, due to the necrosis and apoptosis of cancer cells, tumor-associated antigens (TAAs) and some cytokines such as VEGF, HIF, and FGF will be released into circulation, following an acute antitumor immune reaction and proangiogenic effects, respectively. Finally, immune checkpoint inhibitors (anti-PD1 mAb and anti-CTLA4 mAb) were used in order to remove the inhibition of antitumor effects and enhance the tumor-killing ability of CTLs. Moreover, antiangiogenesis therapy restrains the formation of blood vessels and leads to vascular normalization, which, in turn, synergically increases the infiltrated frequency of CTLs in tumor lesions.

Recently, a novel TATI modality was put forward that consists of TACE, ablation, TKIs, and immunotherapy applied sequentially to patients with advanced HCC. Four patients underwent TACE treatment at the time of disease diagnosis. During follow-up, the patients were treated with microwave ablation to treat residual tumors or recurrences. For tumor control, the TKI apatinib was administered after ablation. If the tumor was stable or resistant to TKI, apatinib was then continued in combination with immunotherapy (camrelizumab). All four patients survived for 17–32 m with no serious adverse effects (Meng et al., 2020). Furthermore, we can speculate about potential mechanisms of this TATI modality. Locoregional therapies potentially not only just destroy primary tumors and decrease tumor burden but may also cause increases in local hypoxia and vascular permeability, inducing immunogenic effects by releasing tumor antigens from dying cancer cells and eliciting damage-associated molecular patterns that facilitate the action of TKIs and ICIs. In turn, TKIs inhibit angiogenesis and blood vessel production through regulating molecular pathways, which are crucial for tumor growth and maintenance, and ICIs stimulate host immune responses that trigger long-lived tumor destruction. In addition, TKIs affect pathways that are also crucial for immune development and function and may optimize antitumor immune responses emerging from immunotherapy (Lanitis et al., 2015). On the other hand, immunotherapy may consolidate the impressive clinical responses from TKIs into long-lasting clinical remission (Vanneman and Dranoff, 2012; Li et al., 2020). Therefore, combined immunotherapy is expected to shift the current paradigm of approaches that represent effective treatment options for advanced HCC (Figure 1).

## Summary and Future Perspective

The optimal outcomes of treatments for advanced HCC are measured in terms of long-term OS, quality of life, and avoidance of major morbidity. Although locoregional and systemic therapies possess unique advantages in controlling tumor growth and improving prognoses, long-term prognoses are still poor. The synergistic effect of combination therapies should be maximized for patients able to tolerate combination treatments. However, there are still many critical questions that remain unanswered. How should the proper patients for combination therapies be selected? When should patients transition between treatment approaches? How should major organ function be protected to ensure smooth treatments? The safety, feasibility, and effectiveness of combined systemic locoregional therapies need to be confirmed by the ongoing research in order to allow for promising prognoses and a better quality of life for patients with advanced HCC.
